# Supplementary material for: Inadequate antibody validation places substantial numbers of animal and human tissue samples at risk of waste
Source: PLoS Biol. 2026 Oct 6;24(10):e3003984. doi: 10.1371/journal.pbio.3003984 (PMC13641334; doi:10.1371/journal.pbio.3003984)
Supplement: Supplementary file 4 [file pbio.3003984.s004.pdf]

S2 Table. Biological sample usage by antibody commercial status and validation evidence

Sample Category	Papers (n)	Animals‡	Human Tissue	Human Primary Cells	Cell Lines
**ALL PAPERS***	766	9,443	5,015	1,215	569
**By antibody commercial status:**					
Discontinued (16 antibodies)	307	3,036	3,910	729	236
Continued (19 antibodies)	459	6,407	1,105	486	333
**By validation evidence†:**					
**WITHOUT validation**	640	8,064	4,424	1,081	400
Using discontinued antibodies	244	2,217	3,437	599	153
Using continued antibodies	396	5,847	987	482	247
**WITH validation**	120	1,339	513	134	168
Using discontinued antibodies	60	815	421	130	83
Using continued antibodies	60	524	92	4	85

Notes:

* Sample numbers were extractable from 766 of 785 publications analysed

† Validation status and sample numbers could be determined for 760 papers

‡ Animal samples include mouse, rat, and other species combined
