# Supplementary material for: Inadequate antibody validation places substantial numbers of animal and human tissue samples at risk of waste
Source: PLoS Biol. 2026 Oct 6;24(10):e3003984. doi: 10.1371/journal.pbio.3003984 (PMC13641334; doi:10.1371/journal.pbio.3003984)
Supplement: Supplementary file 5 [file pbio.3003984.s005.pdf]

S3 Table. Detailed biological sample usage by technique, antibody status, and validation evidence

ANIMAL SAMPLES

Mouse/Rat samples

Category	Papers (n)	Total	WB	IHC/IHC-IF	ICC	FC
All papers (sample numbers identified)	766	8,533	7,145	1,828	477	59
Discontinued antibodies (16)	307	2,856	2,391	365	160	0
Continued antibodies (19)	459	5,677	4,754	1,463	317	59
**Papers with validation & sample data**	760	8,493	7,109	1,824	477	59
**WITHOUT validation**	640	7,253	6,021	1,580	359	59
Discontinued antibodies	244	2,057	1,735	256	66	0
Continued antibodies	396	5,196	4,286	1,324	293	59
**WITH validation**	120	1,240	1,088	244	118	0
Discontinued antibodies	60	795	656	105	94	0
Continued antibodies	60	445	432	139	24	0

Other species samples

Category	Papers (n)	Total	WB	IHC	ICC
All papers (sample numbers identified)	766	910	726	256	132
Discontinued antibodies (16)	307	180	176	0	24
Continued antibodies (19)	459	730	550	256	108
**Papers with validation & sample data**	760	910	726	256	132
**WITHOUT validation**	640	811	627	256	132
Discontinued antibodies	244	160	156	0	24
Continued antibodies	396	651	471	256	108
**WITH validation**	120	99	99	0	0
Discontinued antibodies	60	20	20	0	0
Continued antibodies	60	79	79	0	0

HUMAN SAMPLES

Human tissue samples

Category	Papers (n)	Total	WB	IHC	FC
All papers (sample numbers identified)	766	5,015	1,024	4,120	19
Discontinued antibodies (16)	307	3,910	864	3,194	0
Continued antibodies (19)	459	1,105	160	926	19
**Papers with validation & sample data**	760	4,937	1,024	4,042	19
**WITHOUT validation**	640	4,424	859	3,654	19
Discontinued antibodies	244	3,437	724	2,821	0
Continued antibodies	396	987	135	833	19
**WITH validation**	120	513	165	388	0
Discontinued antibodies	60	421	140	321	0
Continued antibodies	60	92	25	67	0

Human primary cells/serum samples

Category	Papers (n)	Total	WB	FC	ICC	ELISA
All papers (sample numbers identified)	766	1,215	665	366	76	123
Discontinued antibodies (16)	307	729	403	224	57	57
Continued antibodies (19)	459	486	262	142	19	66
**Papers with validation & sample data**	760	1,215	665	366	76	123
**WITHOUT validation**	640	1,081	600	366	52	66
Discontinued antibodies	244	599	342	224	33	0
Continued antibodies	396	482	258	142	19	66
**WITH validation**	120	134	65	0	24	57
Discontinued antibodies	60	130	61	0	24	57
Continued antibodies	60	4	4	0	0	0

CELL LINES

Human cell line samples

Category	Papers (n)	Total	WB	FC	ICC
All papers (sample numbers identified)	766	569	523	5	120
Discontinued antibodies (16)	307	236	216	1	57
Continued antibodies (19)	459	333	307	4	63
**Papers with validation & sample data**	760	568	522	5	119
**WITHOUT validation**	640	400	364	3	76
Discontinued antibodies	244	153	142	0	29
Continued antibodies	396	247	222	3	47
**WITH validation**	120	168	158	2	43
Discontinued antibodies	60	83	74	1	28
Continued antibodies	60	85	84	1	15

VALIDATION METHODS IN PAPERS WITH VALIDATION DATA

Total papers where validation status could be determined: 760 (of 766 papers with extractable sample numbers)

Papers with validation data: 120 (60 used discontinued antibodies)

Papers without validation data: 640 (244 used discontinued antibodies)

Types of validation used in 120 papers with validation data:

Validation method	Number of papers	Notes
Genetic validation: knockout	20	CRISPR or other knockout control. The definitive genetic control
Genetic validation: knockdown	83	siRNA/shRNA knockdown without an accompanying knockout control. A further 6 papers reported both knockdown and knockout and are counted in the knockout row above; 89 papers reported knockdown in total
Overexpression/tagged expression	40	Tagged protein comparison
Independent antibodies	1	Comparison with independent antibody
Mass spectrometry	0	Immunocapture MS validation
Orthogonal validation		Unable to confidently identify

Note: Some papers used more than one validation strategy, so counts do not sum to 120. Knockout and knockdown together account for 103 papers reporting a genetic validation strategy.

Abbreviations: WB = Western blot; IHC = Immunohistochemistry; IHC-IF = Immunohistochemistry-Immunofluorescence; ICC = Immunocytochemistry; FC = Flow cytometry; ELISA = Enzyme-linked immunosorbent assay
